# A regression framework for brain network distance metrics

**DOI:** 10.1162/netn_a_00214

**Published:** 2022-02-01

**Authors:** Chal E. Tomlinson, Paul J. Laurienti, Robert G. Lyday, Sean L. Simpson

**Affiliations:** Department of Biostatistics, University of North Carolina at Chapel Hill, Chapel Hill, NC, USA; Laboratory for Complex Brain Networks, Wake Forest School of Medicine, Winston-Salem, NC, USA; Department of Radiology, Wake Forest School of Medicine, Winston-Salem, NC, USA; Department of Biostatistics and Data Science, Wake Forest School of Medicine, Winston-Salem, NC, USA

**Keywords:** Graph theory, Connectivity, fMRI, Small-world, Neuroimaging, Jaccard, Kolmogorov–Smirnov

## Abstract

Analyzing brain networks has long been a prominent research topic in neuroimaging. However, statistical methods to detect differences between these networks and relate them to phenotypic traits are still sorely needed. Our previous work developed a novel permutation testing framework to detect differences between two groups. Here we advance that work to allow both assessing differences by continuous phenotypes and controlling for confounding variables. To achieve this, we propose an innovative regression framework to relate distances (or similarities) between brain network features to functions of absolute differences in continuous covariates and indicators of difference for categorical variables. We explore several similarity metrics for comparing distances (or similarities) between connection matrices, and adapt several standard methods for estimation and inference within our framework: standard *F* test, *F* test with individual level effects (ILE), feasible generalized least squares (FGLS), and permutation. Via simulation studies, we assess all approaches for estimation and inference while comparing them with existing multivariate distance matrix regression (MDMR) methods. We then illustrate the utility of our framework by analyzing the relationship between fluid intelligence and brain network distances in Human Connectome Project (HCP) data.

## INTRODUCTION

As brain network analyses have become popular in recent years, neuroimaging researchers often face the need to statistically compare brain networks (Simpson et al., 2013a). Many approaches for relating brain networks to clinical outcomes or demographical variables have been developed. Such methods include, but are not limited to, traditional network models (e.g., exponential random graph models; Lehmann et al., 2021; Simpson et al., 2011, 2012), tensor regression works on brain network (e.g., Zhang et al., 2018, 2019), Bayesian approaches (e.g., Dai & Guo, 2017; Wang et al., 2017), statistical learning techniques (Craddock et al., 2015; Varoquaux & Craddock, 2013; Xia et al., 2020), and testing based on distance correlation (Székely et al., 2007; Székely & Rizzo, 2009). Despite the advances made, analysis methods are still needed that enable the comparison of networks while incorporating topological features inherent in each individual’s network. To develop such an analysis, we can exploit the fact that brain networks often exhibit consistent organizations across subjects. For example, a number of studies have reported that nodes with particular characteristics (e.g., high degree) tend to coincide at the same spatial locations across subjects (Hagmann et al., 2008; Hayasaka & Laurienti, 2010; Moussa et al., 2011; van den Heuvel et al., 2008). Although the set of such nodes may not be exactly the same across subjects, there are large areas of overlap (Hagmann et al., 2008; Hayasaka & Laurienti, 2010). Furthermore, our study on network modules, or communities of highly interconnected nodes, indicated that some building blocks of resting-state functional brain networks exhibited remarkable consistency across subjects (Moussa et al., 2012). It has also been shown that such consistent organizations differ under different cognitive states (Deuker et al., 2009; Moussa et al., 2011; Rzucidlo et al., 2013) or in different groups of subjects (Bassett & Bullmore, 2009; Burdette et al., 2010; Liu et al., 2008; Meunier et al., 2009a; Rombouts et al., 2005; Stam et al., 2007; Yuan et al., 2010). Thus, an analysis method sensitive to such differences in spatial locations or patterns can assess network differences across the entire brain (as opposed to univariate edge-by-edge or node-by-node comparisons). Toward this end, in previous work we developed a permutation testing framework that detects whether the spatial location of network features (such as the location of high degree nodes) mapped back into brain space differs between two groups of networks, and whether distributions of topological properties vary by group (Simpson et al., 2013b). Despite the utility of this method, it has two major weaknesses. First, it cannot account for confounding variables. This means that while we can compare maps of hub regions, for example, between two populations, we cannot control for differences in characteristics such as age or education. Second, the method relies on dichotomous grouping to make comparisons. When making comparisons between groups with clear divisions (male vs. female), this is not an issue. However, it is impossible to assess if there is a significant relationship between network hub location and continuous measures, such as intelligence quotient (IQ) score or age.

To address these limitations, we propose an innovative regression framework to relate distances between brain network features to functions of absolute differences in continuous covariates and indicators of difference for categorical variables. We will consider several different types of metrics for establishing distances (i.e., similarity/dissimilarity) between networks. The first type compares degree distributions. We accomplish this by summarizing similarities in nodal cumulative degree distributions across multiple networks with the Kolmogorov–Smirnov statistic (K-S statistic), a measure that quantifies the distance between two cumulative distribution functions (Kolmogorov, 1933; Smirnov, 1948). The second type takes into account consistency of key node sets. We do so by summarizing similarities in node sets across multiple networks with the Jaccard distance (or Jaccard index), a metric that quantifies difference (or similarity) in partitions of a set (Joyce et al., 2010; Meunier et al., 2009b). The third type of metric measures similarity of nodal degree by employing Minkowski or Canberra distances between nodal degree vectors (Lance & Williams, 1966).

Within our regression framework we adapt several methods for estimation and inference: standard *F* test, *F* test with individual level effects (ILE), feasible generalized least squares (FGLS), and permutation. Each observation in the regression framework includes a “distance” between two individuals, so observations that share individuals are correlated. Thus, the standard *F* test is generally not appropriate but presented for comparison. The other methods attempt to deal with this correlation: (a) *F* test with ILE—including fixed individual level effects within the regression, possibly rendering the *F* test valid; (b) mixed model—including random individual level effects; (c) FGLS—proposing an artful way to estimate the covariance matrix (Aitken, 1936); and (d) Permutation—similarity and distance metrics have unknown distributions; thus, a permutation test may be most appropriate (Simpson et al., 2013b). Permutation tests for these models require permuting the residuals, and we will adapt recent methods to implement this approach (Kherad-Pajouh & Renaud, 2010, 2014).

In this paper, we detail our proposed regression framework, and discuss several methods for estimation and inference to be used on a variety of network similarity/dissimilarity metrics. We assess all combinations of methods and metrics within this framework by using simulated fMRI data with known differences in connectivity matrix distributions. We then apply our framework to functional brain networks derived from the HCP dataset to investigate the relationship between fluid intelligence and network distances after accounting for known confounders.

## METHODS

Please note the following notational choices: bold font is used to denote vectors or matrices, *n* = (np2) = number of observations, *n*_*p*_ = number of participants, *n*_*n*_ = number of nodes, *p* = number of covariates (including intercept, if included).

### Step 1: Network Construction

**Figure 1.**
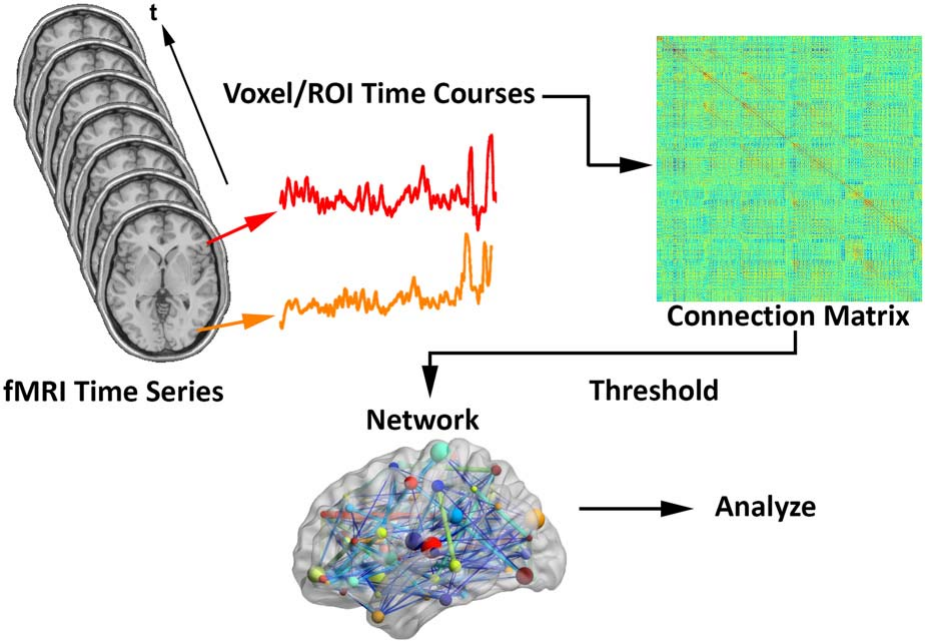
Schematic for generating brain networks from fMRI time series data—recreated from (Fornito et al., 2012; Simpson et al., 2013a). Functional connectivity between brain areas is estimated based on time series pairs to produce a connection matrix. A threshold is commonly applied to the matrix to remove negative and/or “weak” connections.

Assuming fMRI connection matrices have already been obtained (see Figure 1), let ***C***_*i*_ represent a weighted *n*_*n*_ × *n*_*n*_ connection matrix for individual *i*, with matrix entries ranging from 0 (indicating no connection between the respective nodes) to 1 (strongest connection). We only considered undirected networks, so matrices were symmetric, with the *number of row* = *number of columns* = *number of nodes* (methods are easily adaptable if directed networks are desired).

**Figure 2.**
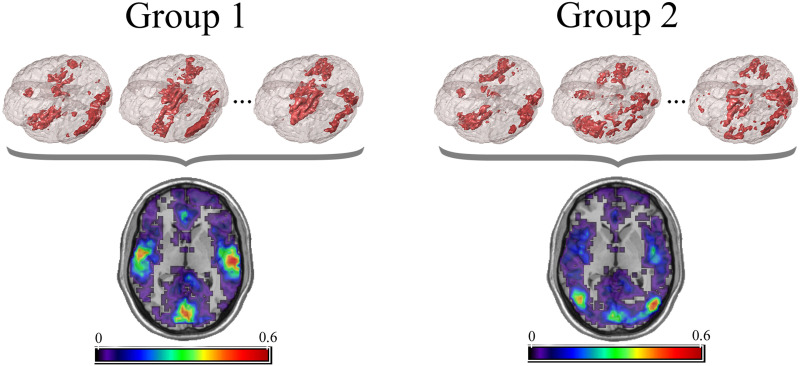
Example visualization of key node sets from voxel-based networks in brain space. The 3D brain images (top) are three representative subjects from each group with the key node sets defined to be those with the top 20% highest degree. Overlap maps (bottom) show the proportion of subjects with key nodes in given areas. Figure recreated from Simpson et al. (2013b).

Let ***b***_*i*_ represent an *n*_*n*_ × 1 binary key node vector for individual *i*, with an entry of 1 representing a key node and 0 a node that is not key. As stated in our previous work (Simpson et al., 2013b), key nodes can be identified based on nodal characteristics such as high degree, high centrality, or other desired characteristics. Since key nodes were compared across subjects, it was important to employ the same criterion in all of the networks (e.g., top 10% highest centrality, node degree >200, etc.). Alternatively, key nodes can be identified as those belonging to a particular module or network community, a collection of highly interconnected nodes. The resulting key nodes would form a set, and the consistency of the spatial location of the nodes could be compared across groups. An example visualization of key node sets from voxel-based networks in brain space is given in Figure 2.

We constructed an *n*_*n*_ × 1 weighted nodal degree vector ***d***_*i*_ by summing ***C***_*i*_ over its rows (or equivalently, columns). Each entry in vector ***d***_*i*_ represented the “degree” of its respective node for individual *i*. It should be noted here that when the binary key node vector has to do with node degree, say 20% highest degree as will used later, ***b***_*i*_ is just a binarized version of ***d***_*i*_.

As was described for the binary key node vector in the last paragraph, other weighted nodal vectors could have been used. For simplicity, we focused on degree within the methods and simulation sections. It should also be noted that none of the methods employed here are specific to nodal vectors. That is, these methods could also be implemented on differences between connection matrices, for example.

### Step 2: Establish Similarity/Dissimilarity Between Networks

This section covers some of the metrics we used to gauge distances between individual networks given the insight they can provide into brain network organizational differences.

#### Kolmogorov–Smirnov (KS) statistic.

Degree distributions, which help quantify the topology of networks, are likely more similar within distinctive groups than they are between these groups. We employed the log of the KS statistic to quantify this potential dissimilarity.

$$\log\left(KS_{ij}\right)=\log\left(sup_{x}\left|F_{i}\left(x\right)-F_{j}\left(x\right)\right|\right)$$

*KS*_*ij*_, a scalar, is the Kolmogorov–Smirnov statistic between nodal degree distributions for individuals *i* and *j*. *F*_*i*_(*x*) represents the empirical distribution function for observations from the nodal degree vector ***d***_*i*_. So, *sup*_*x*_|*F*_*i*_(*x*) − *F*_*j*_(*x*)| gives the biggest difference between the empirical degree distributions between individuals *i* and *j*. Bigger values indicate more dissimilarity.

A note on the logarithmic transformation of the KS statistic: when all distances are nonnegative, it is common practice to take a log transformation. Within our simulations, KS was the only metric that saw improvements in power or type I error when taking such a transformation. For ease of interpretability, none of the other distances presented here utilized a logarithmic transformation.

$${JD}_{ij}=\frac{M_{01}+M_{10}}{M_{11}+M_{01}+M_{10}}$$

#### Jaccard distance.

*JD*_*ij*_, a scalar, is the Jaccard distance (JD) between two (binary) key node vectors of individuals *i* and *j*, ***b***_*i*_, and ***b***_*j*_. *M*_11_ is the number of nodes such that ***b***_*i*_ and ***b***_*j*_ both have a value of 1, *M*_01_ is the number of nodes where ***b***_*i*_ = 0 and ***b***_*j*_ = 1, and *M*_10_ is the number of nodes where ***b***_*i*_ = 1 and ***b***_*j*_ = 0. *JD*_*ij*_ gives the proportion of key nodes (in either set) that do not share key status between individuals *i* and *j*. Values of *JD*_*ij*_ range from 0 (perfect overlap) to 1 (no overlap).

$${JI}_{ij}=1-JD_{ij}=\frac{M_{11}}{M_{11}+M_{01}+M_{10}}$$

#### Jaccard index.

*JI*_*ij*_, a scalar, is the Jaccard index (JI) between two (binary) key node vectors of individuals *i* and *j*, ***b***_*i*_, and ***b***_*j*_. *JI*_*ij*_ gives the proportion of nodes that share key status between individuals *i* and *j*. Values of *JI*_*ij*_ range from 1 (perfect overlap—key node networks that are the same) to 0 (no overlap). Although the JI simply equals 1 − *JD*_*ij*_, it is included here to compare a distance and similarity metric.

$$E_{ij}={\left\|d_{i}-d_{j}\right\|}_{2}={\left(\sum_{k=1}^{\#\text{of}\;\text{nodes}}|d_{i}\left[k\right]-d_{j}\left[k\right]{|}^{2}\right)}^{\frac{1}{2}}$$

#### Euclidean distance.

*E*_*ij*_, a scalar, is the Euclidean distance between nodal degree vector *i* and nodal degree vector *j*, where ***d***_*i*_[*k*] represents the degree of node *k* for individual *i*. Bigger values of Euclidean distance indicate more dissimilarity.

See the Supporting Information for Minkowski distance and Canberra distance.

### Step 3: Evaluating Differences Between Networks

$${\text{Dist}}_{ij}=X_{ij,con}^{T}\beta_{con}+X_{ij,coi}^{T}\beta_{coi}+\varepsilon_{ij}$$

#### Standard *F* test.

*Dist*_*ij*_ represents the distance between nodal vectors of individuals *i* and *j*. *Dist*_*ij*_ is a generic placeholder for any metric outlined previously in Step 2, that is, JD (*JD*_*ij*_), KS statistic (*KS*_*ij*_), Minkowski distance ($M_{ij}^{p}$), or Canberra distance (*C*_*ij*_).

$X_{ij,con}^{T}$, an 1 × (*p* − 1) vector, contains the intercept and differences in confounding covariates (e.g., for our data, sex, educational attainment, age, and body mass index) between individuals *i* and *j* (with corresponding unknown (*p* − 1) × 1 parameter vector ***β***_**con**_) to control for differences that may confound the relationship between the covariate of interest and the given distance.

$X_{ij,coi}^{T}$, a scalar, contains the difference in the covariate of interest (or an indicator of different group membership for group-based analyses) between individuals *i* and *j* (with corresponding unknown parameter *β*_coi_).

Splitting the design matrix ***X***, an *n* × *p* matrix, into confounding and of interest covariates is purely a notational preference. $X_{ij,con}^{T}$ and $X_{ij,coi}^{T}$ can be combined into the 1 × *p* vector $X_{ij}^{T}$ (with corresponding unknown *p* × 1 parameter vector ***β***).

*ε*_*ij*_ accounts for the random error in the distance (Jaccard, KS, etc.) value. If *ε*_*ij*_ were independent, homoscedastic, and approximately normally distributed, the *F* test of a standard linear regression would be an appropriate test. We expect correlation among observations from the same individual, so we included this standard testing procedure here mainly for comparison.

As an example, to test whether there is an association between IQ (continuous) and the spatial consistency of hub nodes (top 20% highest degree) after controlling for age (continuous), sex (binary), and treatment (binary) status, our model would be

$${JD}_{ij}=\beta_{0}+\left|{Age}_{i}-{Age}_{j}\right|\beta_{1}+𝟙\left\{{Sex}_{i}\neq S{ex}_{j}\right\}\beta_{2}+𝟙\left\{{Trt}_{i}\neq {Trt}_{j}\right\}\beta_{3}+\left|{IQ}_{i}-{IQ}_{j}\right|\beta_{4}+\varepsilon_{ij}$$

with the associated null hypothesis *H*_0_: *β*_4_ = 0.

$$\text{Dist}=X^{T}\beta +ID_{1}\alpha_{1}+\ldots +ID_{n_{p}}\alpha_{n_{p}}+\epsilon$$

#### Standard *F* test with individual level fixed effects (*F* test with ILE).

**Dist** is an *n* × 1 vector of known distance metrics (as outlined in Step 2), ***X***^*T*^ is the *n* × *p* design matrix (intercept optional) of known covariates with corresponding *p* × 1 unknown parameter vector ***β***, **ID**_**i**_ is the *n* × 1 known indicator variable for individual *i* with corresponding unknown parameter *α*_*i*_. By accounting for individual level effects, we hope to induce independence, homoscedasticity, and normality for the error terms within the *n* × 1 random vector *ϵ*. This would (potentially) allow for an *F* test to appropriately evaluate the covariates of interest.

#### Mixed model approach.

It should be mentioned that we attempted a mixed model formulation where each individual had their own random effect and all other covariates had fixed effects (where **Dist** = ***X***^*T*^***β*** + **ID**_**1**_b_1_ + ⋯ + **ID**_*n*_*p*__b_*n*_*p*__ + *ϵ* and b_*i*_ ∼ *N*(0, *g*) for all *i*). We also attempted b_*i*_ ∼ *N*(0, *g*_*i*_) for all *i*. Unfortunately, these calculations were too computationally intensive in the simulations we ran (using the lmer function of the R package lme4; Bates et al., 2015). Instead, we tried a generalized least squares approach outlined in the next section.

$$\text{Dist}=X^{T}\beta +\epsilon$$

#### Feasible generalized least squares.

**Dist** and ***X****^T^****β*** (intercept included) are as before, but instead we assume *ϵ* ∼ (**0**, Σ), where Σ is the *n* × n covariance matrix. Generalized least squares (GLS) allows for estimating parameters when there is correlation among the residuals in ordinary least squares regression. However, GLS requires Σ to be known. An unrestricted *n* × n covariance matrix has *n*(*n* + 1)/2 parameters to estimate. This is infeasible as we only have *n* observations. Thus, we restricted the form of Σ in order to estimate it. For a detailed explanation, please see Supporting Information.

#### Permutation test.

A permutation test requires no knowledge of how the test statistic of interest is distributed under the null hypothesis (e.g., *H*_0_: no significant difference among IQ). The distribution under the null hypothesis is empirically “generated” by permuting data labels. We employed the Freedman–Lane approach (Freedman & Lane, 1983; Kherad-Pajouh & Renaud, 2010) using the lmperm function in the R package permuco (Frossard & Renaud, 2019a) with 10,000 permutations, while permuting across individuals to preserve exchangeability. For a detailed explanation, please see Supporting Information as well as the package vignette for permuco (Frossard & Renaud, 2019b).

#### Multivariate distance matrix regression.

Multivariate distance matrix regression (MDMR) is an existing method that has been included here for comparison. It tests the significance of associations of response profile (dis)similarities and a set of predictors. Originally this was done using only permutation (Anderson, 2001), but has been extended to analytic *p* values as well (McArtor et al., 2017). MDMR was run using the mdmr function in the MDMR package in R (McArtor, 2018). Both the permutation and analytic versions were run with the *n*_*p*_ × *n*_*p*_ distance matrix ***D*** (the distance matrix analog of **Dist**) and the *n*_*p*_ × *p* design matrix ***X***_*p*_ (covariates of interest for each participant). The permutation method was run with 10,000 permutations.

## SIMULATION STUDIES

The following simulation study is done using a factorial approach. There are four different settings (named Simulations 1–4). For each simulation setting, we explore four different metrics (KS, JD, JI, and Euclidean). For each simulation and metric combination, six different methods are considered (permutation, *F* test, FGLS, *F* test ILE, MDMR analytic, and MDMR permutation). Subsequent sections will present details and results for each of these “factors.”

### Data

We conducted four simulation settings to assess how well our proposed approaches could detect various relationships between brain network properties and covariates of interest. Each simulation contained 100 subjects, with four covariates of interest. A fair coin was flipped for each subject to determine their sex (*SEX* = male or female). Half of subjects were assigned to treatment and the other half were assigned to placebo (*TRT* = treatment or placebo). IQ and age were both simulated from a normal distribution with mean of 100 and a standard deviation of 15 (rounded to the nearest integer). This resulted in two binary (*SEX*, *TRT*), and two continuous (*AGE*, *IQ*) covariates—variables were given names purely for purposes of explication.

For Simulations 1–3, we simulated fMRI connectivity matrices with 268 nodes each to mimic the experimental data detailed in the next section. In each simulation, a 268 × 268 symmetric matrix (with entries ranging from 0 to 1) was generated for each subject. Entries within each connectivity matrix were drawn from three types of distributions: (a) a low-connectivity noise beta distribution, *Beta*($\frac{4}{3}$, 6); (b) a high-connectivity noise distribution, *Beta*(4, 6); and (c) a signal distribution dependent on covariates and signal percentage, *Beta*(*s*_*p*_ · *a*_*i*_ + (1 − *s*_*p*_) · $\frac{4}{3}$, 6), where *a*_*i*_ = *max*($\frac{4}{3}$, 4 + (*IQ*_1_ − 100) * .2 + (*Trt*_*i*_ = = “*Treatment*”) * 6) represented the covariate-dependent parameter and *s*_*p*_ represented the signal percentage (from 0 to 100%). When the signal percent (*s*_*p*_) was 100%, *Beta*(*s*_*p*_ · *a*_*i*_ + (1 − *s*_*p*_) · $\frac{4}{3}$, 6) = *Beta*(*a*_*i*_, 6). Similarly, when signal percent was 0%, *Beta*(*s*_*p*_ · *a*_*i*_ + (1 − *s*_*p*_) · $\frac{4}{3}$, 6) = *Beta*($\frac{4}{3}$, 6), and was therefore identical to the noise distribution and no longer dependent on covariates.

**Figure 3.**
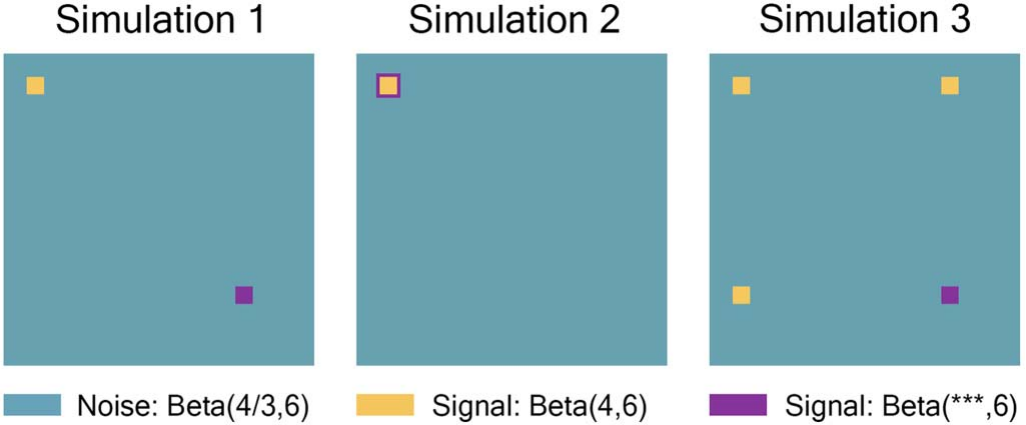
Simulation 1 had a 15 × 15 region where all individuals had the same high-connectivity noise distribution (shown in yellow), a 15 × 15 region where the signal distribution was dependent on covariates (shown in purple), and the rest of the 268 × 268 connection matrix was drawn from the low-connectivity noise distribution (shown in teal). Simulation 2 was the same as 1, but the signal distribution dependent on covariates was moved to expand the border of the 15 × 15 high-connectivity signal distribution to a combined 21 × 21 region. Simulation 3 was the same as 1, with two additional 15 × 15 regions where all individuals had the same high-connectivity distribution. It should be noted that we assumed the connectivity matrices to be symmetric, with 0 entries along the diagonal. The plots were drawn to scale.

Simulation 1 had a 15 × 15 region where all individuals had the same high-connectivity noise distribution, a 15 × 15 region where the signal distribution was dependent on covariates, and the rest of the 268 × 268 connection matrix was drawn from the low-connectivity noise distribution. Simulation 2 was the same as 1, but the signal distribution dependent on covariates was moved to expand the border of the 15 × 15 high-connectivity signal distribution to a combined 21 × 21 region. Simulation 3 was the same as 1, with two additional 15 × 15 regions where all individuals had the same high-connectivity distribution. For a drawn to scale representation of these simulations, see Figure 3. It should be noted that we assumed the connectivity matrices to be symmetric, with 0 entries along the diagonal.

**Figure 4.**
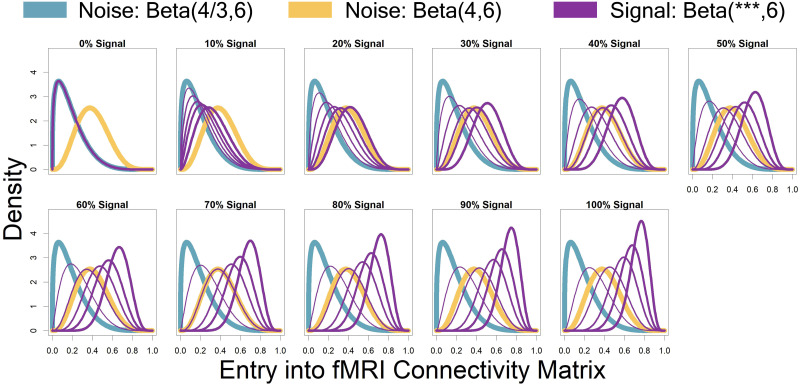
Represented by the same colors from Figure 3 simulated connectivity matrices, displayed here are the distributions used for those matrices as signal percentage increased. The low-connectivity noise distribution was distributed Beta(4/3, 6) and was shown in teal (not affected by signal percentage). The high-connectivity noise distribution was distributed Beta(4, 6) and is in yellow (not affected by signal percentage). The “covariate-dependent” signal region can be seen in purple and was distributed Beta(***, 6). The *** parameter had some distribution based on the underlying covariate distribution and signal percentage. There are five purple distributions in each plot representing the 0.01, 0.25, 0.5, 0.75, and 0.99 quantiles (shown in increasing thickness) from the *** distribution (for example, the 0.25 quantile distribution is represented by an individual with an IQ of 69 and a “Treatment” status or an individual with an IQ of 99 and a “Placebo” status; the 0.75 quantile distribution is represented by an individual with an IQ of 101 and a “Treatment” status or an individual with an IQ of 131 and a “Placebo” status). Furthermore, the “covariate-dependent” (purple) signal region’s distribution goes from being the same as the low-connectivity noise region’s distribution (at 0% signal) to more and more different than the noise region’s distribution as signal percentage increases.

Represented by the same colors from Figure 3 simulated connectivity matrices, Figure 4 displays the distributions used for those matrices as signal percentage increased. The low-connectivity noise distribution was distributed Beta(4/3, 6) and was shown in teal (not affected by signal percentage). The high-connectivity noise distribution was distributed Beta(4, 6) and is in yellow (not affected by signal percentage). The “covariate-dependent” signal region can be seen in purple and was distributed Beta(***, 6). The *** parameter had some distribution based on the underlying covariate distribution and signal percentage. There are five purple distributions in each plot representing the 0.01, 0.25, 0.5, 0.75, and 0.99 quantiles (shown in increasing thickness) from the *** distribution (for example, the 0.25 quantile distribution is represented by an individual with an IQ of 69 and a “Treatment” status or an individual with an IQ of 99 and a “Placebo” status; the 0.75 quantile distribution is represented by an individual with an IQ of 101 and a “Treatment” status or an individual with an IQ of 131 and a “Placebo” status). Furthermore, the “covariate-dependent” (purple) signal region’s distribution goes from being the same as the low-connectivity noise region’s distribution (at 0% signal) to more and more different than the noise region’s distribution as signal percentage increases.

**Figure 5.**
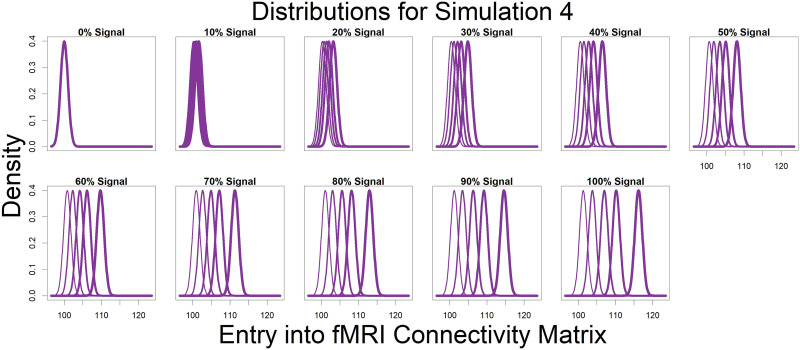
For simulation 4, we simulated nodal degree vectors of length 268 (instead of 268 × 268 connectivity matrices as in Simulations 1–3) to assess the method’s ability to detect distributional differences rather than location differences. All entries of each individual’s degree vector were simulated *Normal*(100 + *s*_*p*_ · *a*_*i*_, 1), where *s*_*p*_ (signal percent) and *a*_*i*_ (covariate-dependent parameter for individual *i*) were as described with Simulations 1–3. There were five purple distributions in each plot, representing the 0.01, 0.25, 0.5, 0.75, and 0.99 quantiles (shown in increasing thickness) from the distribution of the mean parameter, 100 + *s*_*p*_ · *a*_*i*_. At 0% signal, all individual’s nodal degree vectors were drawn from *Normal*(100, 1). As signal percentage increased, the mean parameter of the covariate-dependent distributions became more and more distinct.

For Simulation 4, we simulated nodal degree vectors of length 268 (instead of 268 × 268 connectivity matrices as in Simulations 1–3) to assess the method’s ability to detect distributional differences rather than location differences. All entries of each individual’s degree vector were simulated Normal(100 + *s*_*p*_ · *a*_*i*_, 1), where *s*_*p*_ (signal percent) and *a*_*i*_ (covariate-dependent parameter for individual *i*) were as described with Simulations 1–3. Within Figure 5, there were five purple distributions in each plot, representing the 0.01, 0.25, 0.5, 0.75, and 0.99 quantiles (shown in increasing thickness) from the distribution of the mean parameter, 100 + *s*_*p*_ · *a*_*i*_. At 0% signal, all individual’s nodal degree vectors were drawn from *Normal*(100, 1). As signal percentage increased, the mean parameter of the covariate-dependent distributions became more and more distinct.

### Results

We assessed methods with the four simulation scenarios detailed in the previous section. Each simulation was run 10,000 times. Nodal degree vectors (used for the KS and Euclidean distances) were created by summing across rows of the connectivity matrices. Key nodes of interest (binary degree vectors used for the JD and JI) based on node degree were identified, selecting the top 20% highest degree (hub) nodes and mapping those to 1 while mapping all remaining nodes to 0. The KS statistic and Euclidean distance were calculated for each pair of individuals using their nodal degree vectors. The JD and JI were calculated for each pair of individuals using their binary degree vectors.

**Figure 6.**
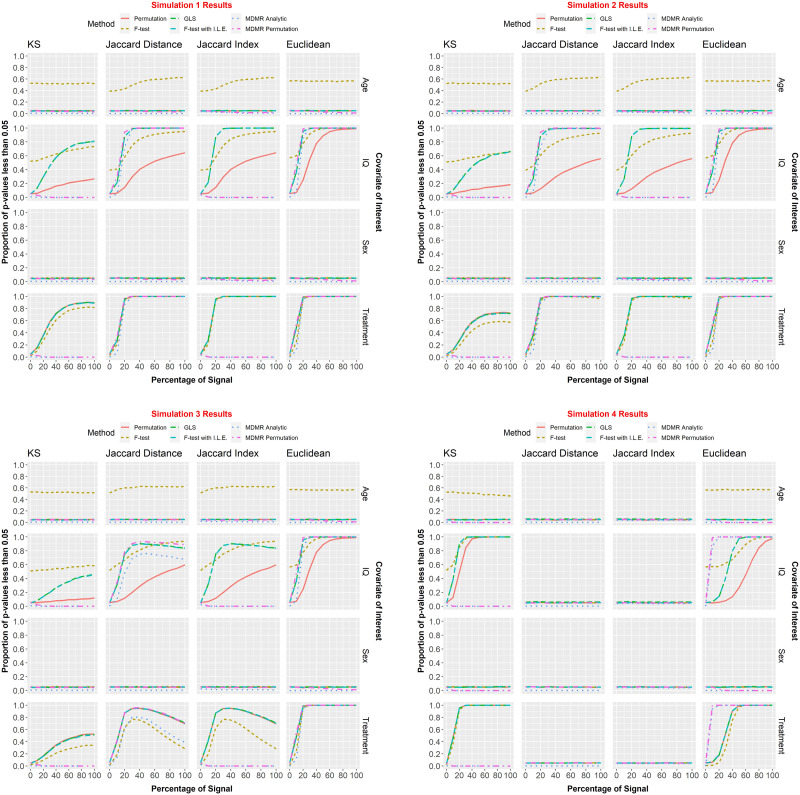
We assessed methods with the four simulation scenarios detailed in the Results section. Each simulation was run 10,000 times. The percentages of *p* values less than *α* = 0.05 for the covariates of interest were recorded for each combination of signal percent (0%, 10%, …, 100%), distance metric (KS, JD, JI, Euclidean), and testing framework (*F* test, permutation, GLS, *F* test with individual level effects, MDMR analytic and MDMR permutation). It should be noted here that Age and Sex are “null” covariates (that have no bearing on the data generating process) and are included to assess type I error control of the methods on both continuous and categorical variables.

The percentages of *p* values less than *α* = 0.05 for the covariates of interest were recorded for each combination of signal percent (0%, 10%, …, 100%), distance metric (KS, JD, JI, Euclidean), and testing framework (*F* test, permutation, GLS, *F* test with individual level effects, MDMR analytic and MDMR permutation). In this section, we discuss whether type I error rate was controlled and at what signal percent the 80% power threshold was reached. Then, we compare results with MDMR. For a visual display of the results, see Figure 6. For more plots and a visual display with additional distance metrics, please see Supporting Information. The standard *F* test did not control type I error when testing age. It was included in the Figure for reference, but is not mentioned any further in this section.

#### Kolmogorov–Smirnov statistic.

For the KS metric, all methods (not including *F* test) adequately controlled type I error. For Simulation 1, the GLS and *F* test with ILE methods reached 80% power within 100% of signal for continuous covariates. The Permutation method never reached 80% power for continuous covariates. For binary covariates, all four methods reached 80% power within 60% of signal. For Simulations 2–3, no methods reached 80% power at any level of covariate-dependent signal. For Simulation 4, the GLS and *F* test with ILE methods reached the power threshold within 20% of signal. Permutation reached the threshold within 30% of signal. All three methods controlled type I error.

#### Jaccard distance and Jaccard index.

Please note, *p* values for all methods used within our regression framework were the same whether the JD or the JI were used (this is not true of the MDMR methods). For the JD and JI, all methods (not including *F* test) adequately controlled type I error. For Simulations 1–3, IQ, the GLS and *F* test with ILE methods reached the power threshold within 20–30% of signal; the permutation method never reached the minimum power requirement. For Simulations 1–3, Treatment, the GLS, *F* test with ILE, and Permutation methods reached the power threshold within 20% of signal. Surprisingly, as signal percentage increased, some situations had decreasing power with some even falling far back below the power threshold. This only occurred with the Jaccard metrics. For Simulation 4, no methods could detect significance. All methods’ power levels hovered around *α* = 0.05, the significance level. This behavior was expected. For a given individual, all entries of the nodal degree vector are drawn independently and identically distributed (iid). Then, highest valued entries of the vector are selected as key nodes. Because all nodes are iid, the distribution set of “key nodes” is uniform on its support. While the centrality parameter of the distribution varies from person to person based on their covariates, this location shift is effectively canceled out by the binarization. Thus, a difference in the mean of the distributions does not lead to a difference in the distribution of the key node vectors, and so it is unsurprising that the power in this setting appears to be 5%. It is not that the Jaccard is causing power issues here, rather, it is because the null is actually true here.

#### Euclidean.

For the Euclidean metric, all methods (not including *F* test) adequately controlled type I error. For Simulations 1–3, IQ, the GLS and *F* test with ILE methods reached the power threshold within 20% signal. The permutation method reached the minimum power requirement within 50% of signal. For Simulations 1–3, Treatment, all methods reached the power threshold within 20% signal. For Simulation 4, IQ, the GLS and *F* test with ILE methods reached the power threshold within 50% signal. The permutation method reached the minimum power requirement within 80% of signal. For Simulation 4, Treatment, all methods reached the power threshold within 40% signal.

#### Comparison with MDMR.

For KS and JI, neither MDMR permutation nor MDMR analytic had adequate power for the KS statistic. Our approaches vastly outperformed them for all simulation scenarios. For Simulations 1–3, JD and Euclidean, the MDMR permutation method had comparable power with the GLS and *F* test with ILE methods, while MDMR analytic had slightly less power. For Simulation 4, Jaccard, see explanation in section JD and JI above. For Simulation 4, Euclidean, both MDMR methods had considerably more power than any of the methods within our framework. MDMR analytic type I error rate remained close to 0 for all simulations. MDMR permutation started off with the expected type I error rate of approximately 0.05, but as signal increased, type I error rate approached 0 as well.

## EXPERIMENTAL STUDIES

### Data

The HCP data released to date include 1,200 individuals (Van Essen et al., 2013). Of those, 1,113 (606 females; 283 minority) have complete MRI images, cognitive testing, and detailed demographic information. The 397 subjects used are what remained after quality control assessment of head motion and global signal changes for both scan types, removal of those with missing data, and random selection of one individual from each family to ensure between-subject independence. Participants in the HCP completed two resting-state scans and two working memory scans. The two scans were collected with different phase encoding (right to left vs. left to right). The resting-state scans were collected back-to-back while participants quietly viewed a fixation point. The 2-back task was a block design that interleaved the 2-back condition with a 0-back condition and a rest period. The working memory task utilized photos and different blocks had different photo types (faces, body parts, houses, and tools). Participants were alerted prior to each block to indicate the task type. For the 2-back they were instructed to respond anytime the current stimulus being presented matched the stimulus two trials back. The HCP performed extensive testing and development to ensure comparable imaging at the two sites (Van Essen et al., 2012). The blood oxygenation level-dependent (BOLD)–weighted images were collected using the following parameters: TR = 720 ms, TE = 33.1 ms, voxel size 2 mm^3^, 72 slices, 1,200 images.

The main covariate of interest for this analysis was fluid intelligence. Other covariates included in model formulation were age, body mass index (BMI), education, handedness, income, alcohol abuse, alcohol dependence, ethnicity, race, sex, and smoking status. For a summarization and explanation of the variables, see Table 1 and Table 2.

**Table 1.** Summarization and explanation of HCP covariates treated as continuous (within the regression framework)

	Mean (*SD*)	Notes
Age	28.7 (3.7)	In years
BMI	26.5 (5.1)	Body mass index
Education	15.0 (1.7)	Integer values 11 to 17 (years pf education completed)
Fluid intelligence	17.3 (4.7)	Integer valued from 4 to 24
Handedness	65.1 (43.6)	Values range from −100 to 100 by 5 (−100, −95, …, 95, 100)
Income	5.0 (2.1)	SSAGA income score - Total household income: <$10,000 = 1, 10K–19,999 = 2, 20K–29,999 = 3, 30K–39,999 = 4, 40K–49,999 = 5, 50K–74,999 = 6, 75K–99,999 = 7, >=100,000 = 8

**Table 2.** Summarization and explanation of HCP covariates treated as categorical (within the regression framework)

Alcohol abuse	**54** met the DSM4 criteria for alcohol abuse, **343** did not
Alcohol dependence	**28** met the DSM4 criteria for alcohol dependence, **369** did not
Ethnicity	**41** Hispanic/Latino, **351** Not Hispanic/Latino, **5** Unknown or Not Reported
Race	**1** Am. Indian/Alaskan Nat., **31** Asian/Nat. Hawaiian/Other Pacific Is., **48** Black or African Am., **13** More than one, **10** Unknown or Not Reported, **294** White
Sex	**207** Female, **190** Male
Smoking status	**69** reported as still smoking, **328** did not

### Data Processing and Network Generation

The current project used the minimally processed fMRI data provided by the HCP (Glasser et al., 2013) for resting-state and working memory. Additional preprocessing steps included motion correction using ICA-AROMA (Pruim et al., 2015), removal of the first 14 volumes from each scan, and band-pass filtering (0.009–0.08 Hz) to remove physiological noise and low-frequency drift. It was necessary to account for the block design of the working memory task. First, the block design was modeled in SPM12, yielding regressors for the 0-back and rest blocks as well as the cues. Additionally, since each scan was collected twice with different phase encoding, the scans were concatenated and a scan-specific regressor was added. All regressors, including the total gray matter, white matter, and cerebrospinal fluid signals and realignment parameters were used in a single regression analysis. The residual signal following regression of the extraneous variables was retained only for periods aligned with the 2-back blocks. The blocks of data were then concatenated into a single time series containing 274 BOLD images. Resting-state data followed a similar pipeline, but without the task-specific regressors. The resulting resting-state data contained 2,372 BOLD images. After preprocessing, the brain was parcellated into 268 regions as defined in the Shen Atlas (Shen et al., 2013), and the signal from all voxels within each region was averaged for each participant. A functional network was constructed for each participant by computing the Pearson (full) correlation between the resultant time series for each region pair. Negative correlation values were set to zero because the neurobiological interpretation of positive and negative edges are very different (Parente et al., 2017; Schwarz & McGonigle, 2011), and since the distributions of network variables (such as degree) are different for positive and negative edges (Fraiman et al., 2009; Saberi et al., 2021). Although negative correlations are not regularly used in network neuroscience, if they are used, positive and negative networks should be generated and assessed separately (Schwarz & McGonigle, 2011). For the current work we focused on positive networks, but one could simply perform an additional analysis on negative networks if so desired.

### Results

Nodal degree vectors (used for the KS and Euclidean distances) were created by summing across rows of the connectivity matrices. Key nodes of interest (binary degree vectors used for the JD) based on node degree were identified, selecting the top 20% highest degree (hub) nodes and mapping those to 1 while mapping all remaining nodes to 0. KS statistic and Euclidean distance were calculated for each pair of individuals using their nodal degree vectors. The JD was calculated for each pair of individuals using their binary degree vectors.

Distance covariates for each pair of individuals were calculated. A continuous variable’s distance (age, for instance) was calculated as |*Age*_*i*_ − *Age*_*j*_| for the pair of individuals *i* and *j*. A binary or categorical variable’s distance (education, for instance) was calculated as 𝟙{*Edu*_i_ ≠ *Edu*_*j*_} for the pair of individuals *i* and *j*.

We evaluated differences between networks using the standard *F* test with ILE, as it was the best in our simulations at controlling type I error and providing sufficient power across all distance metrics (while also being computationally inexpensive). Parameter and standard error estimates can be found in Supporting Information Tables S1 and S2. Each parameter estimate represented the average amount the given brain distance metric (KS, Jaccard, etc.) changed based on a one-unit difference in the respective covariate, after controlling for other covariates. A complete list of *p* values for both resting-state and working memory can be seen in Table 3. Given the high degree of dependence between these results, and the illustrative and exploratory nature of our analysis, there have been no adjustments for multiple comparisons.

**Table 3.**
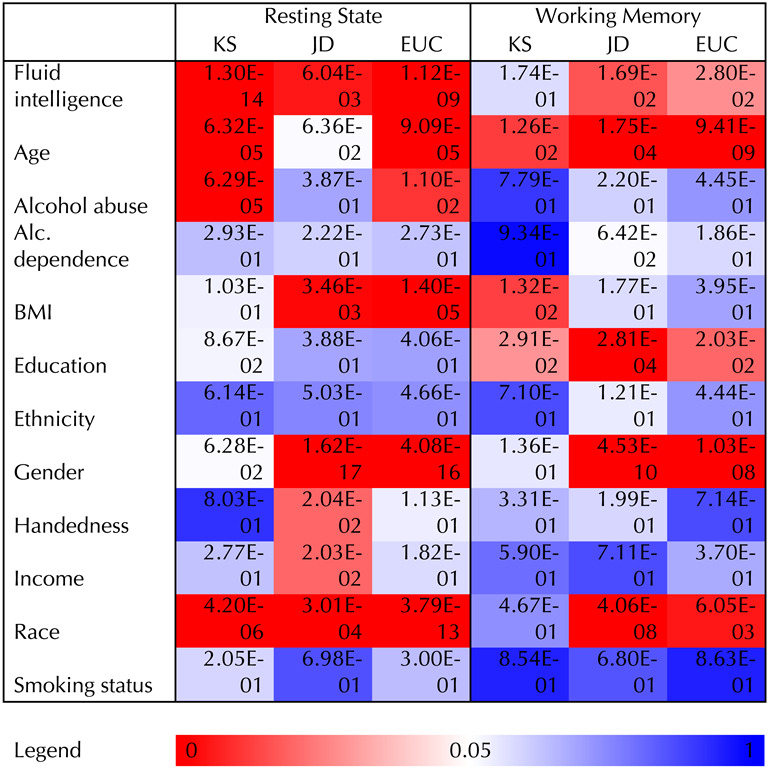
*P* values for HCP resting-state and working memory brain scans when modeled with our given regression framework and tested using the standard *F* test with fixed individual level effects

*Note*. Parameter estimates and standard errors can be found in Supporting Information.

**Figure 7.**
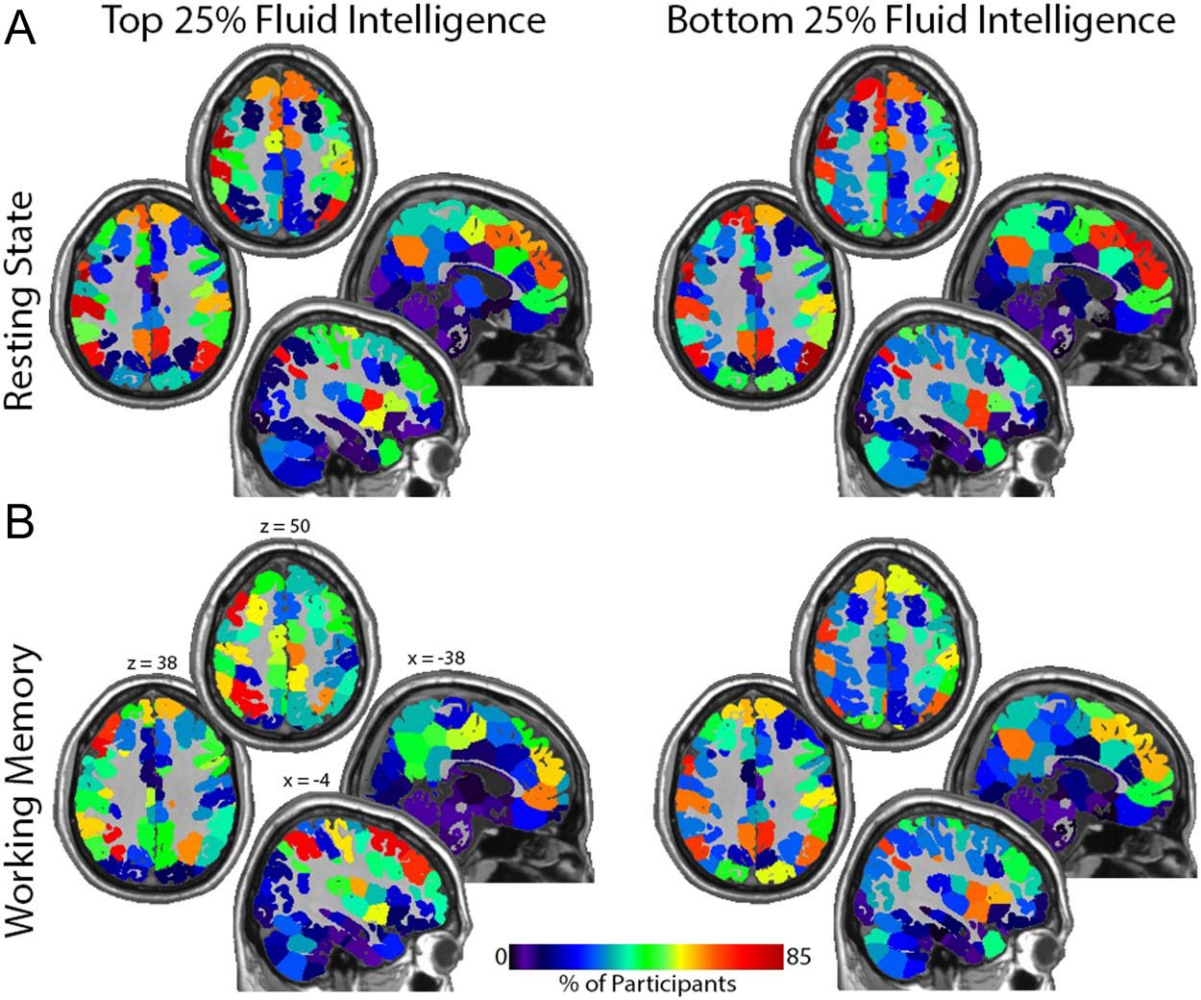
Maps showing the location of network hubs (top 20% degree) for the top and bottom intelligence quartiles. (A) Resting state: note the high incidence of hubs in the medial and lateral parietal cortex and the medial frontal lobe in both groups. These regions are central components of the default mode network. The significant association between degree location and intelligence is demonstrated by the reduction in default mode network hubs with increases in fluid intelligence. (B) Working memory: hubs are more concentrated in the central executive network as intelligence increases. This shift is best appreciated in the top quartile group where hubs are concentrated in lateral rather than medial frontal regions and in more superior parietal areas. Note that the hubs remain fairly localized to the default mode network for the bottom quartile group. Each quadrant shows two axial and two sagittal images. The Montreal Neurological Institute (MNI) coordinates shown in the bottom left quadrant apply to all quadrants. Calibration bar applies to all images.

After adjusting for the other confounding variables, the covariate of interest, fluid intelligence, had a statistically significant relationship with all distance metrics (KS, JD, Euclidean) for resting-state fMRI, and non-KS distance metrics for working memory fMRI. The level of significance was considerably higher for the resting-state data. Figure 7A shows that the resting-state spatial distribution of brain hubs are relatively concentrated in the brain regions making up the default mode network (known to have high degree at rest). However, there is a higher concentration of hubs localized to the default mode network in the bottom quartile of fluid intelligence compared to those in the top quartile. Figure 7B shows that hub locations during the working memory task shift from default mode areas to regions that make up the central executive attention network (CEN) more so in the top quartile of fluid intelligence. Note that in those individuals in the bottom quartile of intelligence, the hubs are most prominent in the areas of the default mode network even during the working memory task, although the magnitude of the overlap is lower. The CEN maps onto what is sometimes referred to as the fronto-parietal network. There is evidence that the density of structural connectivity in that network predicts working memory capacity, with higher capacity individuals exhibiting greater connectivity (Ekman et al., 2016).

Among the confounding variables, some had significant relationships with all distance metrics except for the JD (age and alcohol abuse within resting state). There were also certain variables that were significant for one fMRI task but not the other (alcohol abuse, BMI, and education). Some covariates were significant for all location-specific metrics but not for the distance between distribution comparator KS statistic (BMI – rest; gender – rest and working memory; age, fluid intelligence and race – working memory). This was indicative of a detectable difference in location-specific degree, but not in distribution.

In addition to our primary analysis detailed above, we also examined these relationships with respect to differences in modularity between individuals employing scaled inclusivity, given that modularity analyses can capture the spatial distribution of intrinsic brain networks that are associated with various cognitive tasks (Moussa et al., 2012). The description of this secondary analysis, along with the corresponding results and figures can be found in the Supporting Information.

## DISCUSSION

It is of great interest to compare differences in fMRI connection matrices between individuals by covariates. Our previous work developed a novel permutation testing framework to detect differences between two groups (Simpson et al., 2013b). Here, we advanced that work to allow both assessing differences by continuous phenotypes and controlling for confounding variables. We proposed an innovative regression framework to relate distances between brain network features to functions of absolute differences in continuous covariates and indicators of difference for categorical variables, and explored several similarity metrics for comparing distances between connection matrices. The KS statistic measures how different distributions of topological properties vary between two individuals. Key node metrics (like the JD) quantify how much the spatial location of key brain network regions differs between two networks. The Euclidean norm (along with Canberra, Minkowski, Weighted Jaccard, etc.) measures whether the spatial location of degree-weighted brain network regions differ. Several additional similarity/dissimilarity metrics mentioned below were also assessed, but details were left out for the sake of brevity. The binary network-based metrics left out included the Similarity Matching, Russel-Rao, Kulczynski, and Baroni-Urbani and Buser. Little difference or improvement was found (in Simulations 1–3) for any of these metrics when compared with the commonly used Jaccard. The weighted network-based metrics left out included the Weighted Jaccard, Manhattan, and Maximum distances, with none different or better than the already presented Canberra or Minkowski distances (when tested in Simulations 1–3). Any other distance or similarity metrics could be used. Future work might include (a) testing other metrics, (b) further investigating the behavior of the Jaccard metric, and (c) taking a deeper dive into understanding when and how to choose a distance metric.

Several standard methods for estimation and inference were adapted to fit into our regression framework: standard *F* test, *F* test with ILE, GLS, and permutation (inference only). All combinations of these approaches and the distance metrics were assessed via four simulation scenarios. The KS statistic was found to have low power (relative to the other distance metrics) when testing location-specific differences. However, if interested in comparing distributions, the KS statistic was preferred. The JD did not have consistent or predictable power, and further work should investigate the reasons underlying this. An argument could be made for several different distance metrics when detecting location-specific differences between degree vectors, as they had very similar results. We prefer to use Euclidean distance, as it is commonly recognized and had the best overall (slightly) type I error control and power. Future work could include a further investigation into how and when to choose specific distance metrics. Regarding the comparison of estimation and testing methods (standard *F* test, *F* test with ILE, GLS, permutation), we recommend the *F* test with ILE as it was among the best at controlling type I error and providing sufficient power while also being computationally inexpensive.

An FGLS approach for estimation and inference was proposed in this framework. Although it is slower (computationally) than the standard *F* test with ILE, the FGLS approach performed just as well as the recommended Standard *F* test with ILE (in terms of type I and type II error).

An analysis of the HCP data was completed using the standard *F* test with ILE and several distance metrics. After adjusting for the other confounding variables, the covariate of interest, fluid intelligence, was significantly related with all distance metrics (KS, Jaccard, Euclidean) for both fMRI tasks (resting state, working memory). This analysis suggested the hubs become more strongly concentrated in default mode network regions at rest with decreasing fluid intelligence. As intelligence increases, there are greater shifts in the spatial locations of hubs from the default mode network at rest to the central executive network during a working memory task.

Many existing methods exist for relating network metrics and phenotypes. Such methods include, but are not limited to traditional network models (e.g., exponential random graph models; Lehmann et al., 2021; Simpson et al., 2011, 2012), tensor regression works on brain network (e.g., Zhang et al., 2018, 2019), Bayesian approaches (e.g., Dai & Guo, 2017; Wang et al., 2017), statistical learning techniques (Craddock et al., 2015; Varoquaux & Craddock, 2013; Xia et al., 2020), and testing based on distance correlation (Székely et al., 2007; Székely & Rizzo, 2009). We believe our method most closely relates to MDMR. MDMR tests the significance of associations of response profile (dis)similarities and a set of predictors. Originally this was done using only permutation (Anderson, 2001) but has been extended to analytic *p* values as well (McArtor et al., 2017). Via simulation, a comparison to our regression framework showed that MDMR performs relatively well for the Euclidean and Jaccard distances (as well as other Minkowski distances—see Supporting Information) in terms of power. However, its type I error rate properties were not well understood. For many covariates, close to 0% of tests had *p* values less than 0.05. Further investigation should be done to better understand this property. Furthermore, MDMR was not able to detect differences on either the KS statistic or JI. Our framework vastly outperformed MDMR for these two metrics for all simulation scenarios except for Simulation 4/Jaccard in which the results were comparable (see section *Jaccard distance and Jaccard index* in Results for an explanation).

We have developed a testing framework that detects whether the spatial location of key brain network regions and distributions of topological properties differ by phenotype (continuous and discrete) after controlling for confounding variables in static networks. More generally, this framework allows relating distances between networks (e.g., Jaccard, KS distance) to covariates of interest. Our chosen method, *F* test with ILE, is computationally inexpensive, generally interpretable, and well understood by most scientists. We believe this adds a convenient tool to the neuroscience toolbox. Future work plans to extend this approach by creating a dynamic network analog that uncovers whether within- and across-task time-varying changes in these spatial and distributional patterns differ by phenotype.

## ACKNOWLEDGMENTS

The authors thank Hongtu Zhu, Professor of Biostatistics at University of North Carolina at Chapel Hill, for suggesting that we assess the logarithmic transformations of the distance metrics. We also thank Dale Dagenbach, Professor of Psychology at Wake Forest University, for his insights into interpreting our HCP results regarding IQ.

## SUPPORTING INFORMATION

Supporting information for this article is available at https://doi.org/10.1162/netn_a_00214. Simulation and HCP code is available: https://github.com/applebrownbetty/braindist_regression. HCP data is publicly available for download.

## AUTHOR CONTRIBUTIONS

Chalmer E. Tomlinson: Conceptualization; Data curation; Formal analysis; Investigation; Methodology; Project administration; Resources; Supervision; Validation; Visualization; Writing – original draft; Writing – review & editing. Paul J. Laurienti: Data curation; Resources; Visualization; Writing – original draft; Writing – review & editing. Robert G. Lyday: Data curation; Visualization; Writing – original draft; Writing – review & editing. Sean L. Simpson: Conceptualization; Data curation; Formal analysis; Funding acquisition; Investigation; Methodology; Project administration; Resources; Supervision; Validation; Visualization; Writing – original draft; Writing – review & editing.

## FUNDING INFORMATION

Sean L. Simpson, National Institute of Biomedical Imaging and Bioengineering (https://dx.doi.org/10.13039/100000070), Award ID: R01EB024559. Sean L. Simpson, Wake Forest Clinical and Translational Science Institute, Award ID: NCATS UL1TR001420.

## TECHNICAL TERMS

Permutation testing framework: A method to test statistical significance by permuting labels.
Degree distribution: The probability distribution of the degree of nodes across a network (np2): mathematical notation for “*n*_*p*_ choose 2,” which represents the number of pairs one could choose amongst the *n*_*p*_, number of participants.
Mixed model: Statistical model containing both fixed (population-level) and random (individual-level) effects used to model multivariate data.
Empirical distribution function: Estimate of the cumulative distribution function using the data.
Logarithmic transformation: Taking the log of a variable.
Power: Probability of correctly rejecting the null hypothesis.
Random effect: Variable whose effect varies across individuals.
Fixed effects: Variables whose effects are constant across individuals.
Fair coin: A coin which has a 50% chance of landing on either side.
Symmetric matrix: A matrix which entry *a*_*ij*_ = *a*_*ji*_ for all *i* and *j*. In the connectivity matrix space, this lets us know we are considering an undirected network.
Beta distribution: A family of continuous probability distributions defined on the closed interval [0, 1].
Type I error rate: Probability of incorrectly rejecting the null hypothesis.
